# A Strategy to Detect Emerging Non-Delta SARS-CoV-2 Variants with a Monoclonal Antibody Specific for the N501 Spike Residue

**DOI:** 10.3390/diagnostics11112092

**Published:** 2021-11-12

**Authors:** Rama Devudu Puligedda, Fetweh H. Al-Saleem, Cristoph Wirblich, Chandana Devi Kattala, Marko Jović, Laura Geiszler, Himani Devabhaktuni, Giora Z. Feuerstein, Matthias J. Schnell, Markus Sack, Lawrence L. Livornese, Scott K. Dessain

**Affiliations:** 1 Center for Human Antibody Technology, Lankenau Institute for Medical Research, Wynnewood, PA 19096, USA; puligeddar@mlhs.org (R.D.P.); al-saleemf@mlhs.org (F.H.A.-S.); kattalas@mlhs.org (C.D.K.); himadev2@gmail.com (H.D.); 2 Department of Microbiology and Immunology, Thomas Jefferson University, Philadelphia, PA 19107, USA; christoph.wirblich@jefferson.edu (C.W.); matthias.schnell@jefferson.edu (M.J.S.); 3 Nicoya Lifesciences, Kitchener, ON N2G 2K4, Canada; marko@nicoyalife.com; 4 Department of Internal Medicine, Lankenau Medical Center, Wynnewood, PA 19096, USA; geiszlerl@mlhs.org (L.G.); livornesel@mlhs.org (L.L.L.J.); 5 Debina Diagnostics, Newtown Square, PA 19073, USA; gzfgan083@gmail.com; 6 Pro-SPR, 52477 Alsdorf, Germany; interaction@pro-spr.com

**Keywords:** COVID-19, SARS-CoV-2, delta variant, variants of concern, clinical diagnostic test, monoclonal antibody, OCMS™

## Abstract

Efforts to control SARS-CoV-2 have been challenged by the emergence of variant strains that have important implications for clinical and epidemiological decision making. Four variants of concern (VOCs) have been designated by the Centers for Disease Control and Prevention (CDC), namely, B.1.617.2 (delta), B.1.1.7 (alpha), B.1.351 (beta), and P.1 (gamma), although the last three have been downgraded to variants being monitored (VBMs). VOCs and VBMs have shown increased transmissibility and/or disease severity, resistance to convalescent SARS-CoV-2 immunity and antibody therapeutics, and the potential to evade diagnostic detection. Methods are needed for point-of-care (POC) testing to rapidly identify these variants, protect vulnerable populations, and improve surveillance. Antigen-detection rapid diagnostic tests (Ag-RDTs) are ideal for POC use, but Ag-RDTs that recognize specific variants have not yet been implemented. Here, we describe a mAb (2E8) that is specific for the SARS-CoV-2 spike protein N501 residue. The 2E8 mAb can distinguish the delta VOC from variants with the N501Y meta-signature, which is characterized by convergent mutations that contribute to increased virulence and evasion of host immunity. Among the N501Y-containing mutants formerly designated as VOCs (alpha, beta, and gamma), a previously described mAb, CB6, can distinguish beta from alpha and gamma. When used in a sandwich ELISA, these mAbs sort these important SARS-CoV-2 variants into three diagnostic categories, namely, (1) delta, (2) alpha or gamma, and (3) beta. As delta is currently the predominant variant globally, they will be useful for POC testing to identify N501Y meta-signature variants, protect individuals in high-risk settings, and help detect epidemiological shifts among SARS-CoV-2 variants.

## 1. Introduction

The efforts to control SARS-CoV-2 have been challenged by the emergence of variant strains that have important implications for clinical and epidemiological decision making [1,2]. These variants arise through error-prone genome replication and the outgrowth of strains with mutations that provide a selective advantage. The CDC designated four of these strains variants of concern (VOCs), namely, B.1.1.7 (alpha, United Kingdom), B.1.351 (beta, South Africa), P.1 (gamma, Brazil), and B.1.617.2 (delta, India), although alpha, beta, and gamma have recently been downgraded to variants being monitored (VBMs) [3]. These emerging variants can evade antibody immunity, whether provided by vaccination or passive immunization with monoclonal antibodies (mAbs) [4,5]. They are often more transmissible than earlier strains and warrant enhanced mitigation practices [6,7]. Highly pathogenic variants pose a particular risk to health care facilities, congregate housing settings, public transportation hubs, and high-risk occupational environments.

As multiple variants can circulate simultaneously within a population, variant-specific testing is necessary to effectively diagnose and manage SARS-CoV-2 infections [8] and detect changes in SARS-CoV-2 variant epidemiology [9]. For example, the bamlanivimab/etesevimab mAb combination (Lilly) is not active against the beta and gamma variants, and its use is not authorized when the frequency of mAb-resistant variants exceeds 5% [10].

SARS-CoV-2 variants can be differentiated by amino acid changes in the spike protein receptor-binding domain (RBD) (Table 1), which mediates receptor binding and is one of the major targets of the neutralizing antibody response [4]. One of the most frequent changes is N501Y, which defines a meta-signature of 35 convergent mutations that are associated with increased virulence and evasion of host immunity [11]. The N501 residue lies on the “right shoulder” of the RBD, where it directly contacts the cellular receptor, angiotensin-converting enzyme 2 (ACE2) [12]. N501Y provides a selective advantage over Wuhan-Hu-1 (L) by increasing the affinity of the RBD for ACE2 3- to 16-fold and collaborating with other RBD mutations (especially E484K) to increase binding and infectivity [12,13]. An RBD mutation screen for high-affinity ACE2 binding repeatedly produced *de novo* N501Y mutants, consistent with the observed worldwide appearance of multiple independent N501Y-containing variants [14]. Natural selection of N501Y-containing variants is predicted to increase infectivity in non-immune individuals and enable breakthrough infections in vaccine recipients [10,15].

N501Y is found in the alpha, beta, and gamma variants. Two recently emerged variants further exemplify the threat posed by N501Y-containing SARS-CoV-2 variants, Mu (B.1.621) and C.1.2 [16,17]. Both possess the N501Y and E484K mutations, which provide resistance to vaccine immunity and reduced neutralization by mAb therapeutics [18,19]. They also contain additional spike and non-spike mutations that likely contribute to their enhanced fitness and infectivity [2]. Mu was first identified in Colombia in January 2021 and C.1.2 in South Africa in March 2021. Since then, both have spread globally, including to the United States and Europe.

Variant testing generally relies on nucleic acid amplification tests (NAATs), such as DNA sequencing, S-gene target failure (SGTF), multiplex PCR, and CRISPR [20,21,22]. Some NAATs have been adapted for POC use, with processing times of 30 min or less, but they require specialized equipment for nucleic acid amplification and detection [23]. Immunoassays that detect viral antigens, e.g., antigen-detection rapid diagnostic tests (Ag-RDTs), are technologically well suited to POC testing, as many can be read visually [23]. They can also be used for testing at home [24] and may be ideal in low-resource settings [25]. Ag-RDTs are typically lateral flow assays (LFAs) that can be read within 15 min. They are less sensitive than NAATs, but they have high negative predictive values and are therefore ideal for POC testing, in which rapid turnaround time and high testing frequency are essential for pandemic control [26,27]. Positive Ag-RDT results correlate with high viral loads and the presence of culturable virus, potentially important surrogates of transmissibility [28,29,30]. Over 30 SARS-CoV-2 Ag-RDTs have been granted an emergency use authorization by the FDA [31]. However, variant-specific tests have not yet been implemented [23,26].

The implementation of variant-specific antigen tests can follow an incremental strategy directed at ongoing epidemiological trends. Delta is currently the most prevalent SARS-CoV-2 variant in the United States and globally (https://www.gisaid.org/hcov19-variants/; Accessed on 10 November 2021). Therefore, Ag-RDTs are currently needed to detect infections with emerging non-delta variants that may be vaccine resistant and capable of challenging delta’s dominance. This study is based on a human mAb that binds N501 but not Y501 and can differentiate delta from the (formerly designated) VOCs alpha, beta, and gamma in an ELISA. Furthermore, among the Y501-containing variants, mAb CB6 (the progenitor of etesevimab, Lilly) [32] binds alpha and gamma, but not beta. An Ag-RDT incorporating these two mAbs may be useful for variant-specific SARS-CoV-2 POC testing.

## 2. Materials and Methods

### 2.1. SARS-CoV-2 Spike Antigens and Antibodies

A Wuhan-Hu-1 SARS-CoV-2 spike protein cDNA was cloned into the XhoI and NheI sites of a modified recombinant VSV vector containing an additional transcription start/stop signal between the G and L genes. The recombinant virus was recovered on 293T cells as described previously [33] and filtered through 0.22 µm PVDF filters (MilliporeSigma, Burlington, MA, USA)). The filtered virus was then used to inoculate human BEAS-2B lung cells (gift from R. Plemper, University of Georgia) seeded in Cellstack culture chambers (Corning, Corning, NY, USA). The infected cells were cultured in serum-free Optipro medium (Invitrogen, Waltham, MA, USA). Cell culture supernatant was harvested three days post-inoculation, clarified by centrifugation at 3000 g, and filtered through 0.45 µm PES membrane filters (Nalgene, Rochester, NY, USA). The filtered supernatant was layered on 20% sucrose in DPBS, and particles were sedimented by ultracentrifugation in a SW32 rotor (Beckman, Brea, CA, USA) for 1.5 h at 25,000 rpm. Viral particles were resuspended in phosphate-buffered saline and inactivated with 0.05% beta-propiolactone (BPL). After overnight incubation at 4 °C, the particles were incubated at 37 °C for 45 min to hydrolyze BPL and filtered through 0.22 µm PES filters (MilliporeSigma). To separate the glycoproteins from the ribonucleoprotein complex, 2% beta-octyl-glucopyranoside (OGP) was added to the viral particles. After 15 min incubation at room temperature, the mixture was centrifuged for 1.5 h in a SW55 rotor (Beckman) at 45,000 rpm. After centrifugation, the supernatant was collected and filtered through 0.22 µm PES filters. For protein analysis, 3 µg particles and 1 µg OGP-solubilized glycoproteins were resolved on a denaturing SDS-polyacrylamide gel. (Figure S1). The gel was fixed and stained with SYPRORuby (Thermo Fisher Scientific, Waltham, MA, USA) according to the instructions provided by the manufacturer. Images of the stained gel were acquired on a Fluochem M instrument (Biotechne, Minneapolis, MN, USA).

We also expressed a SARS-CoV-2 S1 domain fragment as a trimeric protein in Expi-293F cells, in part following [34] (Figure S1). We used the original L strain sequence (GenBank: NC_045512) [35] and produced a fusion protein that included residues G283-F718 (eliminating the S1 amino terminal domain and extending to the S1–S2 boundary) and a mutated furin cleavage site. The fusion protein included a mu-phosphatase signal peptide (N-terminal) and a C-terminal fibritin T4 trimerization domain, followed by a Myc site and a 6XHis tag. A gene encoding this fusion protein was produced by Twist Bioscience (South San Francisco, CA, USA) and cloned into the pTwist CMV BetaGlobin expression vector. The construct was transiently transfected into Expi-293F cells (Thermo Fisher) following the manufacturer’s instructions. Due to limited secretion of the protein, on Day 5, we pelleted the cells by centrifugation at 3000 rpm at 4 °C for 20 min. The pellet was resuspended in Takara xTractor™ buffer (Takara Bio, Mountain View, CA, USA) with GenDEPOT Protease Inhibitor Cocktail II, EDTA Free (10X) (Thermo Fisher), incubated on ice with intermittent mixing for 15 min, and then centrifuged at 3500 rpm for 15 min at 4 °C. The supernatant was filtered through a 0.45 μm filter, and the protein was isolated with a Capturem™ His-Tagged Purification Column (Takara). The purity and integrity of S1 trimer were assessed by SDS:PAGE (data not shown) and Western blot (Figure S1) and detected with the Anti-6X His tag^®^ antibody [HIS.H8] (Cat: ab18184; Abcam, Cambridge, MA, USA) and Peroxidase AffiniPure Goat Anti-Mouse IgG, Fcγ fragment specific (RRID: AB_2313585, Jackson ImmunoResearch, West Grove, PA, USA).

### 2.2. Commercial Antigens

The following S1 antigens were obtained from Sino Biological, Chesterbrook, PA: Wuhan-Hu-1 S1 (L): SARS-CoV-2 (2019-nCoV) Spike S1-His Recombinant Protein (Cat: 40591-V08H); Wuhan-Hu-1 S1 with D614G: SARS-CoV-2 (2019-nCoV) Spike S1 (D614G)-His Recombinant Protein (Cat: 40591-V08H3); Wuhan-Hu-1 RBD (L): SARS-CoV-2 (2019-nCoV) Spike RBD-His Recombinant Protein (Cat: 40592-V08H); alpha S1 (B.1.1.7, UK): SARS-CoV-2 (2019-nCoV) Spike S1(HV69-70 deletion, Y144 deletion, N501Y, A570D, D614G, P681H)-His Recombinant Protein (Cat: 40591-V08H12); beta S1 (B.1.351, South Africa), only RBD and D614G changes: SARS-CoV-2 (2019-nCoV) Spike S1(K417N, E484K, N501Y, D614G)-His Recombinant Protein (Cat: 40591-V08H10); beta S1 (B.1.351, South Africa): SARS-CoV-2 (2019-nCoV) Spike S1 (L18F, D80A, D215G, LAL242-244 deletion, R246I, K417N, E484K, N501Y, D614G)-His Recombinant Protein (Cat# 40591-V08H15); gamma RBD (P.1, Brazil/Japan): SARS-CoV-2 (2019-nCoV) Spike RBD (K417T, E484K, N501Y) Protein (His Tag) (Cat: 40592-V08H86); gamma S1 (P.1, Brazil/Japan): SARS-CoV-2 (2019-nCoV) Spike S1 (L18F, T20N, P26S, D138Y, R190S, K417T, E484K, N501Y, D614G, H655Y) Protein (His Tag) (Cat# 40591-V08H14); epsilon S1 (B.1.429, California): SARS-CoV-2 (2019-nCoV) Spike S1 (W152C, L452R, D614G) Protein (His Tag) (Cat: 40591-V08H17); kappa RBD (B.1.617.1, India): SARS-CoV-2 (2019-nCoV) Spike RBD (L452R, E484Q) Protein (His Tag) (Cat# 40592-V08H88); delta RBD (B.1.617.2, India): SARS-CoV-2 Spike RBD (L452R, T478K) Protein (His Tag) (Cat# 40592-V08H90); N501Y (alpha) RBD: SARS-CoV-2 (2019-nCoV) Spike RBD (N501Y)-His Recombinant Protein (Cat# 40592-V08H82); E484K RBD: SARS-CoV-2 (2019-nCoV) Spike RBD(E484K)-His Recombinant Protein (Cat# 40592-V08H84); K417N RBD: SARS-CoV-2 (2019-nCoV) Spike RBD (K417N)-His Recombinant Protein (Cat# 40592-V08H59); Wuhan-Hu-1 S1 (L) biotinylated: SARS-CoV-2 (2019-nCoV) Spike S1-His Recombinant Protein, Biotinylated (Cat: 40591-V08H-B).

### 2.3. Discovery of the 2E8 Human Monoclonal Antibody

We collected sera and peripheral blood mononuclear cells (PBMCs) from 25 patients at least 14 days following complete recovery from a SARS-CoV-2 infection. All subjects provided signed informed consent under a protocol approved by the Main Line Hospitals Institutional Review Board. We assayed the sera for immunoreactivity with the SARS-CoV-2 S1 pseudotyped VSV (VSV G:S1) particles by ELISA. A male volunteer in his 50s was found to have anti-spike titers >1:8000. He was a Caucasian, without comorbid health conditions, diagnosed by RT-PCR testing in New York City in March 2020. He required hospitalization due to respiratory decompensation but was not intubated. Blood was sampled 25 days after his last COVID-19-related symptom. We fused CD27+ peripheral blood mononuclear cells and the LCX OCMS fusion partner cell line [36]. Hybridomas were screened for binding to the VSV G:S1 particles by ELISA. A positive well was subjected to three rounds of single-cell cloning to isolate a monoclonal cell expressing the anti-SARS CoV-2 mAb, 2E8. For scale up, the hybridoma was adapted to 5% Ultra Low IgG FBS (Thermo Fisher), and the mAb was isolated from the supernatant using a Pierce™ Protein G column (Cat: 89927; Thermo Fisher). The 6A control mAb was also produced from its hybridoma [37]. The 2E8 Ig variable domains were amplified by RT-PCR, using the Qiagen RNA extraction kit (Cat: 74124; Qiagen, Germantown, MD, USA), and reverse transcribed with the Omniscript RT Kit (Cat: 205111; Qiagen). Variable domain cDNA sequences were amplified with consensus primer sets specific for human immunoglobulin heavy and light chain genes [38]. Amplified sequences were isolated by agarose gel electrophoresis, purified with the QiaQuick Gel Extraction kit (Cat: 28706; Qiagen), sequenced by Psomagen, Inc. (Rockville, MD, USA), and analyzed with IMGT/V-QUEST [39]. We also isolated polyclonal IgG from plasma of the individual who provided the 2E8 mAb, using a Pierce™ Protein G column (Thermo Fisher).

### 2.4. Production of Recombinant Antibodies

For recombinant 2E8 production, full-length human IgG1 and Igλ cDNAs encoding the 2E8 mAb heavy and light chain variable regions were produced and subcloned into pTwist CMV BetaGlobin expression plasmids (Twist Bioscience) [40]. The plasmids were transiently transfected into Expi-293F cells following the manufacturer’s instructions. On day 4, cell culture supernatants were harvested and purified with the Pierce™ Protein G column (Thermo Fisher). Purity and size were confirmed by SDS:PAGE (data not shown). The 2E8 mAb concentration was measured with a NanoDrop1000 (Thermo Fisher). Recombinant human mAbs CB6 [32], CR3022 [41], and 4G1 [42] were produced by the same method.

### 2.5. Surface Plasmon Resonance (SPR) Spectroscopy

The binding kinetics of the 2E8 IgG with the SARS-CoV-2 S1 protein was determined using the 2-channel OpenSPR (Nicoya Lifesciences, Kitchener, ON). The assays were performed at 21 °C with buffer PBS 0.05% Tween-20 (PBST). The S1 protein (Wuhan-Hu-1 S1 (L): SARS-CoV-2 (2019-nCoV) Spike S1-His Recombinant Protein (Cat: 40591-V08H)) was immobilized on a nitrilotriacetic acid (NTA) sensor chip following EDTA conditioning. His-streptavidin (Abcam, Cat: ab78833) was immobilized in the reference channel as a control ligand. Purified recombinant 2E8 mAb was diluted in PBST supplemented with 0.1% BSA and injected for 5 min at a flow rate of 20 μL/min in a concentration series from 1.23 nM to 100 nM, with 10 min dissociation time. Sensors were regenerated with two injections of 500mM imidazole per regeneration step, with 40 s contact time and 270 s dissociation time. Sensorgrams were fitted with TraceDrawer analysis software (Ridgeview Instruments, Uppsala, Sweden).

### 2.6. ELISAs

Recombinant antibody binding to SARS-CoV-2 spike antigens: NUNC high-binding ELISA plates (Thermo Fisher) were coated in PBS with 500 ng/well antigen at 4 °C overnight. Plates were washed three times with PBS containing 0.05% Tween-20 (PBST) and blocked with blocking buffer (BB) (PBST containing 5% non-fat dry milk) at 37 °C for one hour. Ten-fold serial dilutions of the mAbs were diluted in BB, added in triplicate, and incubated for 1 h at 37 °C. Following 3 washes with PBST, samples were incubated with horseradish peroxidase (HRP)-conjugated mouse anti-human IgG Fc fragment specific secondary antibody (RRID AB_2687484; Southern Biotech, Birmingham, AL, USA), diluted 1:1500 in BB, for one hour at 37 °C. After three washes, the plate was incubated with OPD substrate (P8287; Sigma Aldrich, St. Louis, MO, USA) for 10 min at RT. The reaction was stopped with 1N HCL, and the optical density (OD) at 490 nm was read with a Biotek Synergy II microplate reader (BioTek Instruments, Winooski, VT, USA). The mouse mAb positive control was SARS-CoV-2 (2019-nCoV) Spike Neutralizing Antibody (RRID:AB_2857934; Sino Biological) and was detected with Goat Anti-Mouse Ig, Human ads-HRP (Cat: 1010-05; Southern Biotech).

Sandwich ELISAs for specific variant binding: NUNC high-binding ELISA plates were coated with 100 ng/well 2E8 or CB6 or 500 ng/well anti-6X His tag^®^ antibody [HIS.H8] (RRID:AB_444306; Abcam), in PBS overnight at 4 °C. Plates were washed and blocked as above. S1 or RBD proteins were added to the plates (500 ng/well) and incubated for 1 h at 37 °C, followed by 3 washes. Biotinylated mouse anti-S1 (RRID:AB_2857934; Sino Biological), biotinylated with EZ-Link Sulfo-NHS-Biotin, (Thermo Fisher), was added (500 ng/well) and incubated for 1 h at 37 °C followed by 3 washes. Pierce Streptavidin-HRP substrate (Thermo Fisher) was added at 1:2000 dilution and incubated for 1 h at 37 °C and washed as above, and then the plates were washed and detected with OPD substrate as above.

### 2.7. Flow Cytometry-Based Receptor-Binding Inhibition Assay

Antibody interference of S1 binding to human ACE2 receptor on the cell surface of 293T cells was measured by flow cytometry. Briefly, 0.1 μg/mL biotinylated SARS-CoV-2 spike S1 (Cat: 40591-V08H-B; Sino Biological) was incubated with 1 μg/mL recombinant mAb or a human ACE2-Fc fusion protein (Cat fc-hace2: Invivogen, San Diego, CA, USA) at 37 ºC for one hour. The S1:mAb mixtures were added to 5 × 105 293T-hsACE2 cells (Cat: C-HA101; Integral Molecular) and incubated for 30 min at room temperature. Following incubation, cells were washed twice with PBS containing 2% fetal bovine serum (PBSF) and incubated with Alexa Flour 488 Streptavidin (RRID: AB_2337249; Jackson ImmunoResearch) (1:200 dilution) to detect S1 binding and Goat Anti-Human IgG (H + L) Antibody, Alexa Fluor 647 Conjugated (RRID:AB_2535862; Thermo Fisher) to detect human IgG binding. After 30 min incubation, cells were washed twice with PBSF and analyzed using a BD FACS Canto II (Becton Dickson, Franklin Lakes, NJ, USA). Data were analyzed using FlowJo 10.6.1. software (Tree Star, Ashland, OR, USA).

### 2.8. Pseudotyped SARS-CoV-2 Antibody Neutralization Assay

The antibody neutralization assay was obtained from Integral Molecular and performed following their protocol, using the 293T-hsACE2 cell line (Cat: C-HA101; Integral Molecular, Philadelphia, PA, USA) and the pseudotyped SARS-CoV-2 (Wuhan-Hu-1 strain) reporter viral particles (RVPs) with luciferase (Cat: RVP-701L, Lot CL-114B, Integral Molecular). Briefly, in a 96-well plate, 5-fold serially diluted mAbs were combined with 10 μL RVPs and incubated for 1 h at 37 °C. Following incubation, 2 × 104 293T-hsACE2 cells were added to each well, mixed gently by pipetting, and then incubated at 37° C with 5% CO_2_. After 72 h, SARS-CoV-2 RVP infection was quantified using the Renilla-Glo^®^ Luciferase Assay System (Cat: E2710, Promega, Madison, WI, USA). Briefly, we centrifuged the plate for 5 min at 2000 rpm, aspirated the supernatants, and added 30 μL PBS to each well, followed by 30 µL Renilla-Glo^®^ Assay Substrate (1:100 dilution). After 10 min, relative luminescence values were measured using the Synergy 2 plate reader (BioTek Instruments). The values from the negative control wells were normalized and used to calculate the percent infection for each concentration. All samples were run in triplicate.

### 2.9. Epitope Binning

We performed competitive binding assays to test whether biotinylated 2E8 could bind SARS-CoV-2 spike antigens (S1 and RBD) in the presence of the human mAbs CB6, CR3022, and the murine SARS-CoV-2 (2019-nCoV) Spike Neutralizing Antibody (RRID:AB_2857934; Sino Biological) [43,44]. Black NUNC MaxiSorP 96-well plates (Thermo Fisher) were incubated overnight with 500 ng/well S1 or RBD, then washed three times with PBST, and blocked with BB for 1 h at 37 °C. The 2E8 was biotinylated with the EZ-Link Sulfo-NHS-Biotin kit (Thermo Fisher), and the S1 and sRBD antigen binding curves were found to be linear between 2.5 pg/mL and 2.5 μg/mL. In the experiments shown, 500 ng/well of the competing mAb was added to half of the antigen wells and PBS to the other half and then incubated for 1 h at 37 °C, followed by 3 PBST washes. The 2E8 serial dilution was added to the entire plate. After three more washes, Pierce Streptavidin-HRP substrate (Thermo Fisher) was added at 1:2000 dilution and incubated for 1 h at 37 °C. Following 3 PBST washes, SuperSignal ELISA Femto Substrate (Thermo Fisher) was added (1:1 ratio), and relative luminescence values were measured using the Synergy 2 plate reader (BioTek). Duplicate binding curves were plotted, and the linear portions were used for analysis.

## 3. Results

### 3.1. A Human mAb That Neutralizes SARS-CoV-2 through Spike RBD Binding

**Figure 1:**
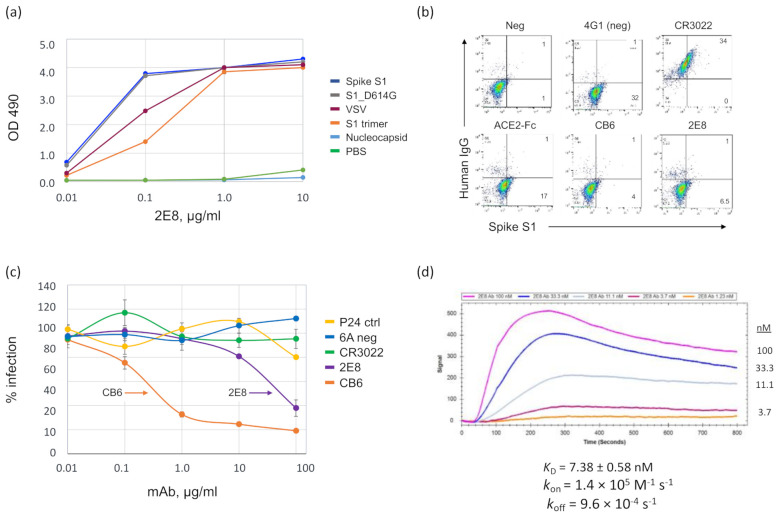
SARS-CoV-2 L strain (Wuhan-Hu-1) spike binding by the 2E8 human mAb. (**a**) The 2E8 mAb binding in a direct ELISA to SARS-CoV-2 antigens: S1, S1 D614G, and nucleocapsid (Sino Biologicals); spike-pseudotyped VSV particles; and a recombinant S1 trimer. Samples were tested in triplicate. Error bars = S.E.M. (not visible due to minimal differences). (**b**) S1 binding to 293T-hsACE2 cells in the presence of 2E8, 4G1 (isotype control IgG), CR3022, CB6, and an ACE2-Fc fusion protein was assessed by flow cytometry. S1 cell binding, *x*-axis; IgG binding, *y*-axis. (**c**) Pseudovirus neutralization assay. 293T-hsACE2 cells were transduced with SARS-CoV-2 luciferase (Wuhan-Hu-1 strain) reporter viral particles (RVPs) in the presence of the mAbs, 6A (isotype control IgG), CR3022, 2E8, and CB6, and a polyclonal IgG isolated from the 2E8 B cell donor (P24). Normalized percent infection is shown; samples were tested in triplicate. Error bars = S.E.M. (**d**) SPR analysis of 2E8 binding to the spike S1 domain, performed on the Nicoya OpenSPR™; KD = 7.38 ± 0.58 nM.

The 2E8 mAb was cloned from a male in his 50s who had a confirmed case of COVID-19 contracted in New York City in March 2020. A peripheral blood sample was obtained 42 days after his first symptom. The 2E8 mAb was cloned using the human hybridoma method described previously [36]. As antigens, we used VSV-G:S1 particles (pseudotyped with the SARS-CoV-2 spike protein S1 domain) and a trimeric, S1 protein (S1 trimer) (Figure S1), both based on the reference sequence Wuhan-Hu-1 (L, NC_045512) [35]. We tested the hybridoma-expressed mAb for binding to commercial antigens (spike S1, spike D614G, and nucleocapsid) and the VSV-G:S1. In a direct ELISA (Figure 1a), the 2E8 bound all four S1 antigens, with somewhat less binding to the VSV-G:S1 and the S1 trimer at the 0.1 µg/mL level (Figure 1a). We then made a recombinant IgG1 2E8 molecule, which was used for all subsequent studies, as well as recombinant IgG1 mAbs, CB6 and CR3022 [32,41].

The 2E8 inhibited the binding of S1 to the 293T-hsACE2 cell line, reducing the amount of right-shifted cells from ~33% to 7.5%, compared to the 4G1 isotype control (Figure 1b). The CB6 mAb also inhibited S1 binding (5%), whereas the CR3022 did not inhibit S1 binding despite adhering to S1 at the cell surface. To test viral neutralization, we used a reporter viral particle (RVP) assay, in which 293T-hsACE2 cells were incubated with SARS-CoV-2 (Wuhan-Hu-1 strain) pseudotyped virions that contain a luciferase transgene (Figure 1c). Cells were infected in triplicate. The data were normalized to the negative control wells and calculated as the % infection. The 2E8 substantially reduced luciferase expression (80% reduction at 10 µg/mL) compared to the 6A non-binding control mAb, the non-neutralizing CR3022 mAb, and a polyclonal IgG (P24) from the B cell donor sampled for 2E8 mAb cloning. However, its activity was 100-fold less than that of the CB6 mAb (85% reduction at 0.1 µg/mL). We measured the binding kinetics of the 2E8 with the Wuhan-Hu-1 S1 using the OpenSPR™ Benchtop SPR System (Nicoya Lifesciences, Kitchener, ON, Canada). The Kinetic analysis is shown in Figure 1d: *K*_D_ = 7.4 ± 0.58 nM, *k*_on_ = 1.4 × 10^5^ M^−1^ s^−1^ ± 2.0 × 10^3^ M^−1^ s^−1^, *k*_off_ = 9.6 × 10^−4^ ± 5.6× 10^−5^ s^−1^ (Figure 1d). This is approximately a three-fold lower affinity than that of the CD6 mAb, 2.49 ± 1.65 nM [32]. The full immobilization sequence of the SARS-CoV-2 S1 domain and 2E8 binding to the immobilized S1 domain are shown in Figures S2 and S3, respectively.

**Figure 2:**
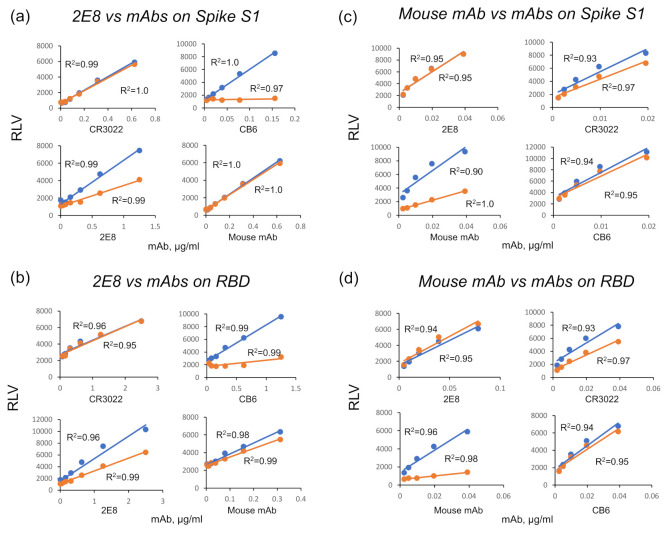
Epitope binning the 2E8 on the SARS-CoV-2 S1 and RBD. We performed competition binding assays to test 2E8 binding in the presence of the CR3022, CB6, and the murine anti-S1 mAb. L-type spike (**a**,**c**) or RBD (**b**,**d**) was captured on the plate and binding of biotinylated 2E8, or the anti-spike murine mAb was tested in the presence of non-biotinylated competitor mAbs. Blue, no competitor; orange, competitor present.

To gain more insight into the nature of S1 binding, we epitope binned the 2E8 in comparison to the CB6 and CR3022 mAbs and a neutralizing, pan-specific anti-spike mouse mAb (Sino Biologicals, RRID: AB_2857934) on the Wuhan-Hu-1 S1 and RBD proteins. We used a binding assay that analyzes mAb competition at the linear portion of an antigen binding curve [43,44]. The 2E8 epitope on S1 and RBD clearly overlaps with CB6 but not CR3022 or the mouse mAb (Figure 2a,b). This is consistent with the observations that CB6 binds S1 in a configuration that overlies the N501 residue and that CB6 and CR3022 bind non-overlapping epitopes [32,41]. Murine mAb binding was not inhibited by 2E8 or CB6, suggesting that it can be used in combination with these mAbs in a SARS-CoV-2 sandwich ELISA (Figure 2c,d).

### 3.2. Binding of 2E8 to Important SARS-CoV-2 Variants

**Figure 3:**
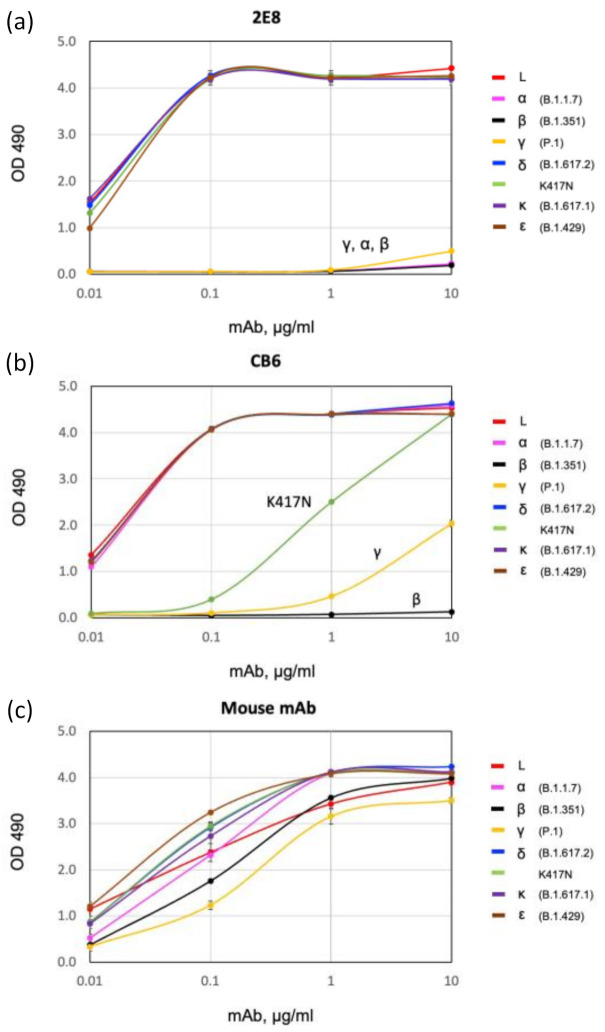
Differential recognition of SARS-CoV-2 variant spike antigen by direct ELISA. The 2E8 (**a**), CB6 (**b**), and the mouse anti-S1 mAb (**c**) were tested for binding to spike antigens adhered to an ELISA plate: L RBD (Wuhan-Hu-1), α S1 (B.1.1.7), β S1 (B.1.351), γ S1 (P.1), δ RBD (B.1.617.2), K417N RBD, κ RBD (B.1.617.1), ε S1 (B.1.429). Error bars = S.E.M.

Present and previously designated VOCs contain mutations in the RBD that can affect binding by mAbs. We used a direct ELISA to compare the binding of 2E8 and CB6 to recombinant S1 and RBD proteins, including S1 proteins corresponding to L, alpha, beta, gamma, and epsilon (B.1.429; L452R, E484Q) (Figure 3). We also tested RBDs corresponding to delta, kappa (B.1.617.1; L452R, E484Q), and the single mutant K417N. The 2E8 bound all the spike proteins tested, except for alpha, beta, and gamma (Figure 3a). As these VBMs share the N501Y RBD mutation, and the alpha contains only the N501Y mutation, we also tested 2E8 binding to an N501Y RBD; no binding was observed (data not shown). In contrast, 2E8 binding to RBDs with a single K417N or E484K mutation was not impaired (Figure 3a). This suggests that the 2E8 interacts with N501 but not substantially with E484 or K417. Equivalent binding to delta, kappa, and epsilon S1 proteins further indicates that 2E8 does not recognize L452 or T478. This indicates that the 2E8 can distinguish between N501 and N501Y independent of changes affecting class I (K417) and class II (E484) neutralizing antibodies. 

CB6 is a well characterized, potent neutralizing mAb that shows reduced neutralization activity against many VBMs. CB6 shows no binding to beta and reduced binding to gamma but no reduction in binding to alpha, delta, kappa, or epsilon (Figure 3b). We explored the difference between binding to the beta and gamma RBDs, which differ only at K417 (beta, K417N; gamma, K417T). K417N by itself significantly reduces binding (Figure 3b). This is consistent with the observations of others [45] and suggests that CB6 is useful to differentiate N501Y-containing variants alpha and gamma from beta in an ELISA. The murine mAb bound to every antigen tested, indicating its suitability as a capture mAb in variant-specific sandwich ELISAs (Figure 3c).

### 3.3. Spike Variant Binding in a Sandwich ELISA

**Figure 4:**
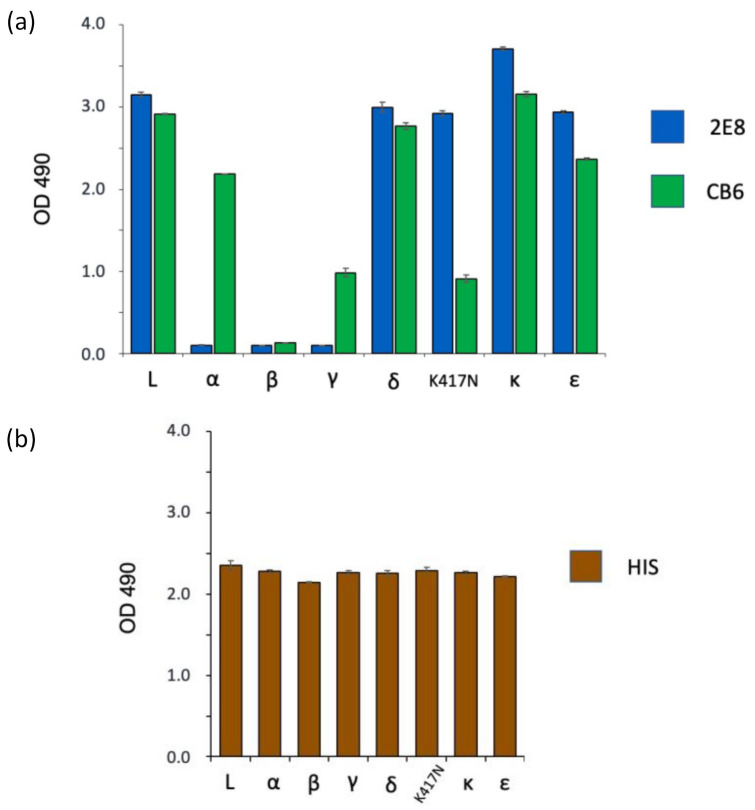
Differential recognition of SARS-CoV-2 variant spike antigens using a sandwich ELISA with human mAbs. The mAbs were adhered to the ELISA plate and tested for capture of soluble spike antigen. Bound antigen was detected with the mouse anti-S1 mAb. (**a**) Both 2E8 and CB6 are compared. (**b**) An anti-His tag capture antibody was used as a positive control. Antigens used (same as Figure 3): L RBD (Wuhan-Hu-1), α S1 (B.1.1.7), β S1 (B.1.351), γ S1 (P.1), δ RBD (B.1.617.2), K417N RBD, κ RBD (B.1.617.1), ε S1 (B.1.429).

Sandwich ELISAs can be used to evaluate mAbs for use in LFAs, as both formats use a pair of non-overlapping mAbs for antigen capture and detection. We tested 2E8 and CB6 binding to variants in a sandwich ELISA format, including an anti-6X His tag antibody (Abcam, Cat: ab18184) as a positive control for antigen capture. The mAbs were used to capture the spike antigens, which were then detected with the biotinylated murine mAb (Figure 4a). We tested binding to the same antigens tested in Figure 3. The 2E8 ELISA bound to L, delta, the K417N mutant, kappa, and epsilon but not to any of the Y501-containing variant proteins: alpha, beta, and gamma. CB6 bound every antigen except beta. As in the direct ELISA, reduced CB6 binding was seen with the gamma and K417N RBDs. The Anti-6X His tag antibody gave an equivalent signal with all antigens. These results are summarized in Table 2. Taken together, these results confirm the utility of the 2E8 mAb to differentiate variants by distinguishing N501 from Y501 in the spike RBD. They further show that CB6 can be used in this format to differentiate Y501-containing VBMs alpha and gamma from beta.

## 4. Discussion

The emergence of SARS-CoV-2 variants has greatly complicated the efforts to control and treat the disease. The variants differ in their ability to evade antibody immunity provided by vaccination or passive immunization and, therefore, may dramatically impact health care facilities, congregate housing settings, public transportation hubs, and high-risk occupational environments. Variant-specific POC testing is necessary to protect individuals in these settings, as well as to screen populations for shifts in SARS-CoV-2 epidemiology. This study advances the concept of using variant-specific Ag-RDTs as a component of the SARS-CoV-2 screening and diagnostic paradigm.

Detection of the N501Y spike mutation with the 2E8 mAb is an efficient way distinguish delta from variants with the N501Y meta-signature [11], such as beta, gamma, mu, C.1.2, and novel N501Y-containing variants yet to emerge. The N501Y mutation has originated independently within multiple viral lineages and is positively selected because it increases infectivity through enhanced ACE2 binding [11,12]. Mutagenesis and modeling experiments suggest that the N501Y mutation will support the evolution of SARS-CoV-2 variants with increased infectivity and resistance to vaccines and therapeutics [13,45]. As the delta variant is currently the dominant strain globally, an Ag-RDT to detect infections with N501Y meta-signature variants will be a powerful tool for disease monitoring and control.

The N501 residue lies on the “right shoulder” of the RBD and directly interacts with ACE2 during cell binding [11,12]. The N501Y mutation does not dramatically alter the overall RBD structure. This suggests that 2E8 binds N501 and/or may be sterically inhibited by Y501. The 2E8 binding site overlaps the CB6 site, yet its binding is not affected by changes at K417, and CB6 binding is largely insensitive to the N501Y change [12]. Furthermore, 2E8 has ~100-fold less neutralizing activity than that of CB6, even though its affinity is only 3-fold lower than that of the CB6 (7.38 ± 0.58 nM vs. 2.49 ± 1.65 nM) [32]. These data highlight the structural independence of these two epitopes in variant RBDs.

The CB6 interaction with the spike has been defined using X-ray crystallography [32]. CB6 is a type I neutralizing mAb that contacts the K417 and N501 residues. CB6 neutralization is unaffected by the N501Y RBD mutation, consistent with the relative unimportance of this residue to CB6 binding. In contrast, the gamma and beta variant changes essentially eliminate CB6 binding and neutralization [46,47,48]. Published data show that CB6 binding affinities for the beta and gamma variants are 42.6-fold and 18.7-fold lower, respectively, in comparison to the wild-type spike. This is attributable to the K417N mutation, which alone reduces affinity 21.9-fold compared to a 13.8-fold reduction from the K417T change. Our results are consistent with these data, which explain how CB6 distinguishes beta from alpha and gamma in our ELISAs.

The 2E8 and CB6 mAbs provide qualitatively different information, because the 2E8 is a poorly neutralizing non-clinical antibody, whereas the CB6 is part of the etesevimab/bamlanivimab therapeutics. A lack of 2E8 binding to a clinical sample suggests additional mutations associated with an increased risk of breakthrough infections and treatment failures. In contrast, a lack of CB6 binding strongly suggests that either the beta or gamma variant is present; furthermore, it predicts resistance to etesevimab. In the current delta-dominant milieu, either finding would ideally trigger a follow-up characterization using an NAAT test.

This study supports a paradigm for the detection of SARS-CoV-2 variants using an Ag-RDT with variant-specific mAbs. The mAb capture/detection pairs used here should be readily adaptable for use in LFAs. Ideally, the 2E8 mAb would be used in a multiplexed assay in parallel with a mAb or ACE2 reagents capable of binding all variants [49,50,51]. Such tests can be an important adjunct to NAATs, as they are ideal for POC testing to protect vulnerable populations and broaden epidemiological surveillance. Both 2E8 and CB6 have immediate applicability for testing while delta is the most prevalent variant. However, additional mAbs will be needed as the variant landscape evolves. This objective should be achievable, as the repertoire of potential variant-specific mutations is well defined, a large number of anti-spike mAbs have been cloned, and extensive structural data describing mAb–spike interactions have been generated.

## 5. Conclusions

It is essential to remain vigilant for emerging SARS-CoV-2 variants that are resistant to pre-existing anti-viral immunity and can out-compete the delta variant. This study of the 2E8 mAb supports an immediately applicable paradigm for the detection of important SARS-CoV-2 variants using an Ag-RDT. Such tests are ideal for POC testing to protect vulnerable populations, plan medical care, and broaden epidemiological surveillance.

## 6. Patents

A patent application has been filed by the Lankenau Institute for Medical Research, which claims the 2E8 mAb and the methods described.

## Figures and Tables

**Table 1:** Major circulating SARS-CoV-2 variants and their RBD mutations.

Variant	Lineage	CDC Classification	RBD Mutation(s)
L	Wuhan-Hu-1	Wild type	N/A
alpha	B.1.1.7	VBM	N501Y
beta	B.1.351	VBM	K417N, E484K, N501Y
gamma	P.1	VBM	K417T, E484K, N501Y
delta	B.1.617.2	VOC	L452R, T478K
kappa	B.1.617.1	VBM	L452R, E484Q
epsilon	B.1.429	VBM	L452R

VBM, variant being monitored; VOC, variant of concern; RBD, SARS-CoV-2 receptor-binding domain; N/A, not applicable.

**Table 2:** The binding of 2E8 and CB6 to SARS-CoV-2 spike proteins.

Variant	Lineage	Tested	RBD Mutation(s)	2E8	CB6
L	Wuhan-Hu-1	RBD	N/A	++	++
alpha	B.1.1.7	S1	N501Y	-	++
beta	B.1.351	S1	K417N, E484K, N501Y	-	-
gamma	P.1	S1	K417T, E484K, N501Y	-	+
delta	B.1.617.2	RBD	L452R, T478K	++	++
kappa	B.1.617.1	RBD	L452R, E484Q	++	++
epsilon	B.1.429	S1	L452R	++	++
N/A	N/A	RBD	K417N	++	+

RBD, receptor-binding domain; S1, spike S1 domain; -, no binding; +, intermediate binding; ++ high binding.

## Data Availability

The DNA sequences for the variable domains of the 2E8 mAb will be available in CoV-AbDab: http://opig.stats.ox.ac.uk/webapps/coronavirus (Accessed on 11 November 2021).

## Supplementary Materials

The following are available online at https://www.mdpi.com/article/10.3390/diagnostics11112092/s1, Figure S1: Pseudotyped VSV-G:S1 and S1 trimer antigens. Figure S2: Full immobilization sequence of the SARS-CoV-2 S1 domain. Figure S3: Anti-spike antibody 2E8 binding to the immobilized S1 domain.
