# Supplementary material for: A Strategy to Detect Emerging Non-Delta SARS-CoV-2 Variants with a Monoclonal Antibody Specific for the N501 Spike Residue
Source: Diagnostics (Basel). 2021 Nov 12;11(11):2092. doi: 10.3390/diagnostics11112092 (PMC8625484; doi:10.3390/diagnostics11112092)
Supplement: Supplementary file 1 [file diagnostics-11-02092-s001.zip › diagnostics-1453327-supplementary.pdf]

Supplementary Information
Puligedda et al.

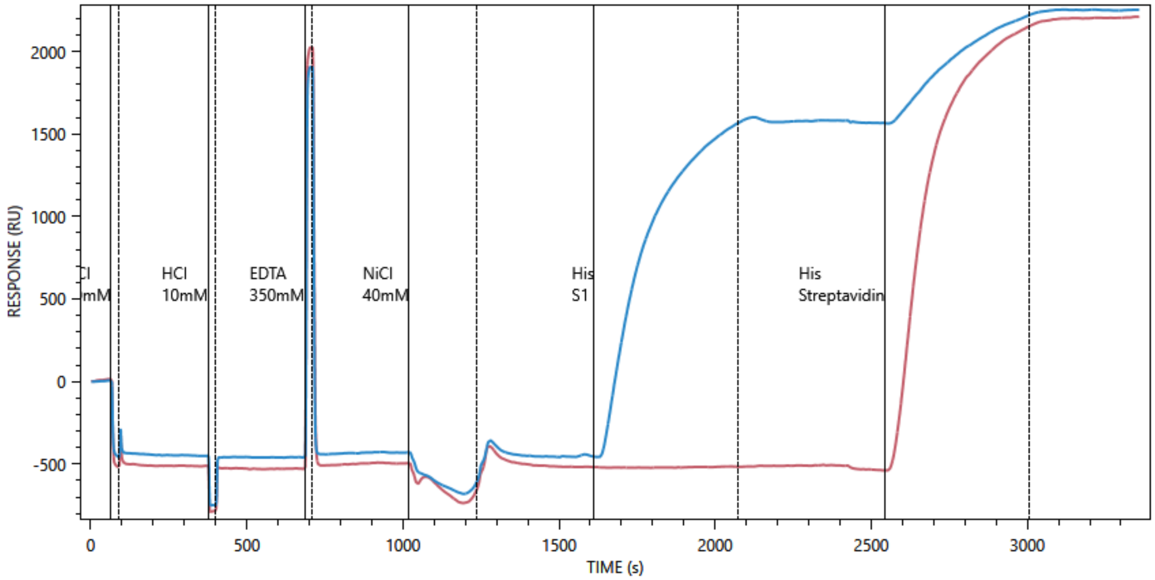

Supplementary Figure 1. Full immobilization sequence of the SARS-CoV-2 S1 domain. The antigen was adhered via a His-tag onto an NTA Sensor in Active (Channel 2). A control His-Streptavidin was used Reference Channel 1. 20 ug/ml for each protein resulted in matching levels of ligand density on the sensor surface at the end of immobilization.

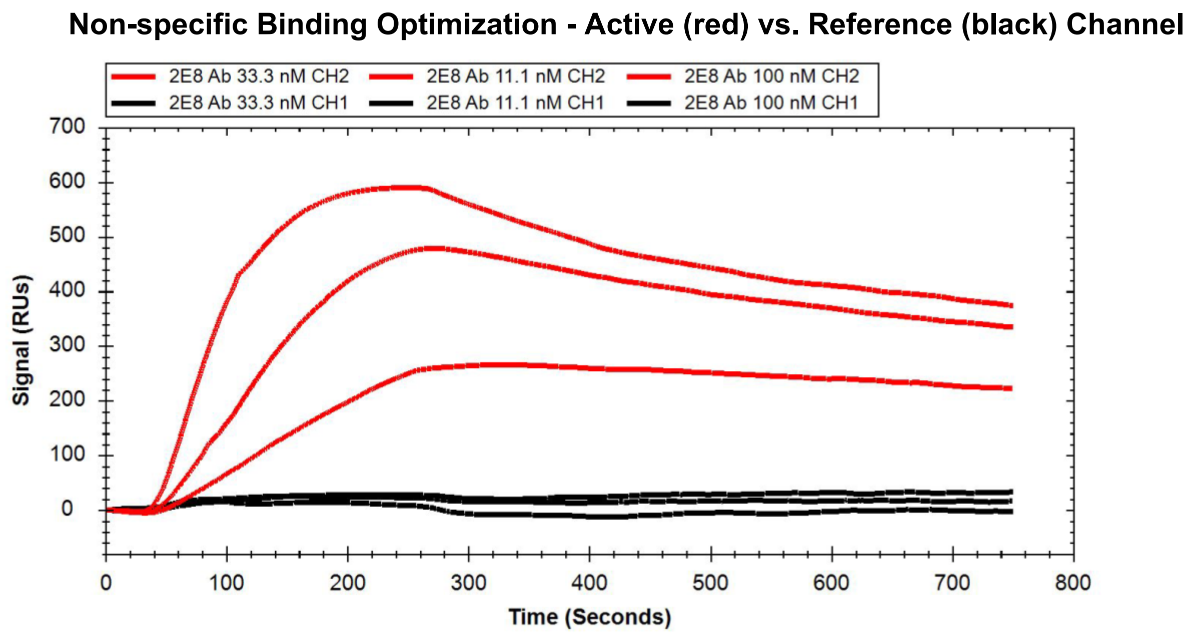

Supplementary Figure 2. Anti-spike antibody 2E8 binding to the immobilized S1 domain. The initial non-specific binding (NSB) test on the NTA sensor is performed with PBST supplemented with 0.1% BSA to reduce non-specific binding. Decreasing concentrations are displayed, starting with the highest concentration at the top.

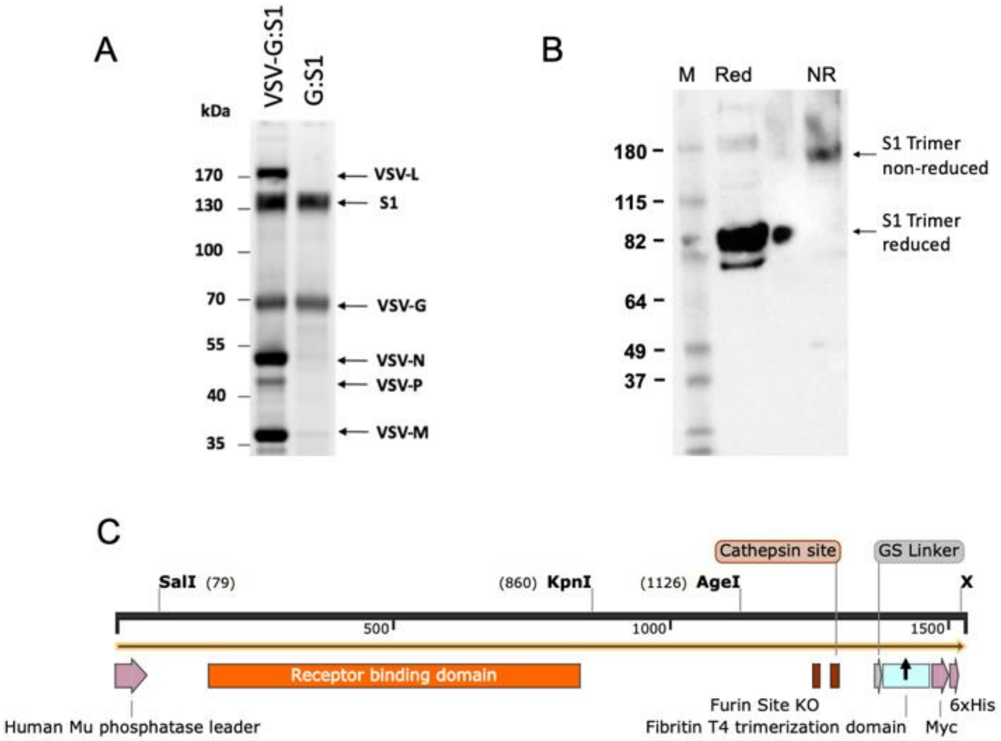

Supplementary Figure 3. Pseudotyped VSV-G:S1 and S1 trimer antigens. (A) SDS:PAGE demonstrating proteins contained in the VSV-G:S1 pseudotyped particles. Detergent-solubilized G:Sa protein is shown for comparison. (B) SDS:PAGE of the S1 trimer, analyzed as reduced (Red) and non-reduced (NR) samples. (C) Map of the gene encoding the S1 trimer.
